# Sphingomyelin synthase overexpression increases cholesterol accumulation and decreases cholesterol secretion in liver cells

**DOI:** 10.1186/1476-511X-10-46

**Published:** 2011-03-21

**Authors:** Nianlong Yan, Tingbo Ding, Jibin Dong, Yue Li, Manping Wu

**Affiliations:** 1 School of Pharmacy, Fudan University, Shanghai, People's Republic of China

## Abstract

**Background:**

Studies have shown that plasma high density lipoprotein cholesterol levels are negatively correlated with the development of atherosclerosis, whereas epidemiological studies have also shown that plasma sphingomyelin level is an independent risk factor for atherosclerosis.

**Methods:**

To evaluate the relationship between cellular sphingomyelin level and cholesterol metabolism, we created two cell lines that overexpressed sphingomyelin synthase 1 or 2 (SMS1 or SMS2), using the Tet-off expression system.

**Results:**

We found that SMS1 or SMS2 overexpression in Huh7 cells, a human hepatoma cell line, significantly increased the levels of intracellular sphingomyelin, cholesterol, and apolipoprotein A-I and decreased levels of apolipoprotein A-I and cholesterol in the cell culture medium, implying a defect in both processes.

**Conclusions:**

Our findings indicate that the manipulation of sphingomyelin synthase activity could influence the metabolism of sphingomyelin, cholesterol and apolipoprotein A-I.

## Background

Atherosclerosis (AS) is the main cause of cardiovascular disease and stroke and a prevalent chronic disease. The metabolism of cholesterol in vivo is tightly associated with AS [1]. Studies have shown that levels of high density lipoprotein cholesterol (HDL-C) in the plasma were negatively correlated with atherosclerosis, whereas increasing plasma HDL-C levels could reduce the development of AS [2]. The mechanism of this effect may be due to high density lipoprotein (HDL) mediated reverse cholesterol transport (RCT), a process used to remove excess cholesterol from peripheral tissues to the liver for excretion. Many lipids and protein molecules are involved in RCT [3], of which Apolipoprotein A-I (Apo A-I) and membrane sphingomyelin are two.

Apo A-I is synthesized in the liver and intestine and is a major protein component of HDL. Apo A-I can serves as an acceptor for phospholipids and cholesterol effluxed from peripheral tissues through the ATP binding cassette transporter A1 (ABCA1) to form nascent HDL particles [4,5]. The nascent particles continue to accept the cholesterol effluxed through ATP binding cassette transporter G1 (ABCG1) to form mature HDL [3,6]. Scavenger receptor BI (SR-BI), an HDL receptor on liver cell membranes, can mediate selective uptake of HDL lipid, and cholesterol can be taken up into the liver and excreted into bile [3,7].

Significant evidence has been presented to confirm the existence of lipid rafts in membranes enriched with sphingolipids and cholesterol in the liquid-ordered phase. Sphingomyelin (SM) is a major component of sphingolipids [8]. Studies have shown that ABCA1, ABCG1 and SR-BI are associated with lipid rafts [9-11]. Therefore, changes in SM biosynthesis could have an impact on lipoprotein metabolism and membrane proteins (ABCA1, ABCG1 and SR-BI), thus influencing lipoprotein and cholesterol levels in the circulation.

Epidemiological investigations have shown that an increased plasma SM levels is an independent risk factor for AS. Recent studies have found that SM accumulates in atherosclerotic plaques [12,13]. Because SM might affect the activity of lecithin cholesterol acyltransferase (LCAT) and lipoprotein lipase (LPL), the inhibition of SM biosynthesis might reduce lipoprotein SM content and improve cholesterol distribution in lipoproteins by enhancing RCT [14].

Serine palmitoyltransferase (SPT) and sphingomyelin synthase (SMS) are two critical enzymes that play important roles in the multienzymatic steps of SM biosynthesis. Pharmacologic inhibition of SPT caused a significant decrease of plasma SM levels and AS plague size in mice [15,16]. SMS is the last critical enzyme for SM biosynthesis and has two isoforms (SMS1 and SMS2). SMS1 is located on the cis-, medial-Golgi apparatus, and SMS2 is found in plasma membranes [17,18]. Our laboratory has found that SMS overexpression increased SM levels in cells and animals [19,20].

In this study, we investigated the effect of SMS overexpression on cholesterol accumulation and secretion in Huh7 cells.

## Materials and methods

### Preparation of cell lines stably overexpressing SMS1 or SMS2

A Tet-off system was used to establish the Huh7 cell lines overexpressing SMS1 or SMS2. Briefly, Huh7 cells were transfected with the regulator plasmid of pTet-Off Vector (Clontech) (to express the tTA protein), using Lipofectamine 2000 (Invitrogen) according to the manufacturer's instructions. G418 (250 μg/ml) was used to select positive clones (named as Huh7-tTA), which were further transfected with Tet-Off-SMS1-FLAG or Tet-Off-SMS2-FLAG (SMS1 and SMS2 expression vectors were purchased from Open Biosystems, USA). The cells were screened under the pressure of Hygromycin B (250 μg/ml, Amresco) and G418 (250 μg/ml). The monoclonal cell lines that survived under the pressure selection were chosen and named SMS1 or SMS2 cells.

### RT-PCR analysis

Total RNA was isolated from cells with Trizol (Takara). Two μg of total RNA was reverse-transcribed and the cDNA was amplified by PCR using a kit (Fermentas). The PCR products were visualized by electrophoresis on 1.5% agarose gels. β-Actin RNA was used as an internal control. The primers (Invitrogen) used for the analyses were the following:

SMS1 sense (5' - GGCTTCTCAGCGTAGTTGGA -3');

SMS2 sense (5'-TATTCGCCTCGTCACTTCTGG-3');

common antisense is FLAG (5'-TCATCGTCATCCTTGTAATCG-3');

β-actin sense (5'-GGGTCACCCACACTGTGCCCATCTA-3');

β-actin antisense (5'- GCATTTGCGGTGGACGATGGAGG-3');

Apo A-I sense (5'-CTCTTCCTGACGGGGAGC-3');

Apo A-I antisense (5'- TCACCTCCTCCAGATCCTTG -3');

ABCA1 sense (5'-AGTACCCCAGCCTGGAACTT-3');

ABCA1 antisense (5'-CTGTCCTTGGCCAGCTTTAG-3');

ABCG1 sense (5'-GAAGGTCTTGAGCAACTCCG-3');

ABCG1 antisense (5'-CAGTAGGCCACTGGGAACAT-3');

SR-BI sense (5'-CAACAACTCCGACTCTGGGCTCT-3');

SR-BI antisense (5'-GTCAGCGTTGAGGAAGTGAGGAT-3');

HMG-CoA reductase sense (5'- GGAGTGGCAGGACCCCTTTGC -3');

HMG-CoA reductase antisense (5'- CCAGCCATGGCAGAGCCCAC -3').

The product lengths of SMS1, SMS2, β-actin, Apo A-I, ABCA1, ABCG1, SR-BI and HMG-CoA reductase are 367 bp, 431 bp, 650 bp, 310 bp, 586 bp, 207 bp, 421 bp and 617 bp, respectively.

### Western blot analysis

Equal amounts of cell homogenates (20 μg protein/lane) were separated on 10% polyacrylamide gels under denaturing conditions. Proteins were electroblotted to a nitrocellulose membrane that had been incubated with respective antibodies: FLAG (CST), Apo A-I (Epitomics), ABCA1 (Boster), ABCG1 (Boster), or SR-BI (Epitomics). The loading control was glyceraldehyde-3-phosphate dehydrogenase. The Super Signal West detection kit (Pierce) was used for the detection step. The maximum intensity of each band was measured using Image-Pro Plus version 6.0 software (Media Cybernetics, Inc.).

### Sphingomyelin synthase activity assay

Cells were homogenized in a buffer containing 50 mM Tris-HCl, 1 mM EDTA, 5% sucrose, and protease inhibitors. The homogenate was centrifuged at 5000 rpm for 10 min, and the supernatant was used to analyze SMS activity. The reaction system contained 50 mM Tris-HCl (pH 7.4), 25 mM KCl, C_6_-NBD-ceramide (0.1 mg/mL) (Invitrogen-MP), and PC (0.01 mg/mL). The mixture was incubated at 37°C for 2 hours. Lipids were extracted in chloroform:methanol (2:1), dried under nitrogen gas, and separated using thin layer chromatography (TLC) [21]. The plate was scanned with a PhosphorImager (Molecular Dynamics; Sunnyvale, CA) and the intensity of each band was measured using Image-Pro Plus version 6.0 software (Media Cybernetics, Inc.).

### Lysenin treatment and cell mortality measurement

Cells were washed twice in PBS and incubated with lysenin (200 ng/ml) for 1 hr. Cell viability was measured using the WST-1 cell proliferation reagent according to the manufacturer's instructions (Roche) [22]

### Analysis of Apo A- I in the medium by ELISA

ELISA analysis of the Apo A-I in the cell culture medium was done as previously reported [23].

### Sphingomyelin and cholesterol measurement

The cell monolayer was washed twice with PBS, and cellular lipids were extracted using n-hexane-2/propanol (3:2). Aliquots of the extracted lipids were subjected to analysis. The SM content was measured by a method previously described [15], and cholesterol was measured using a commercial available kit (Rongshen).

### Statistical Analysis

Data are reported as the mean ± standard deviation (SD). Statistical analysis was performed using Student's t test and one-way ANOVA followed by Tukey's test. Differences were considered significant at *P *< 0.05.

## Results

**Figure 1:**
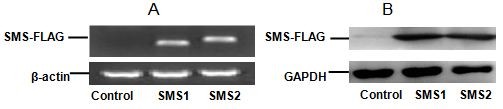
**Detection of SMS-FLAG in control (Huh7-tTA), SMS1 and SMS2 overexpressing cells by RT-PCR (A) and Western blot (B)**.

**Figure 2:**
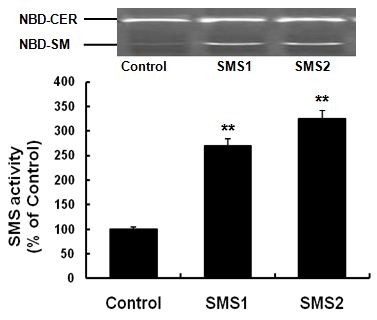
**SMS activity assay**. Values shown are means ± SD (*n *= 6), ** *P *< 0.001 compared to control (Huh7-tTA).

To investigate the ex vivo role of SMS1 and SMS2, we utilized the Tet-off system to express SMS1-FLAG and SMS2-FLAG in Huh 7 cells. RT-PCR of RNA prepared from the cells indicated that both SMS1-FLAG and SMS2-FLAG were expressed (Figure 1A), and this expression was confirmed by Western blot analysis (Figure 1B). Furthermore, SMS overexpression caused a significant increase in total SMS activity compared to controls (Figure 2; *P *< 0.001).

**Figure 3:**
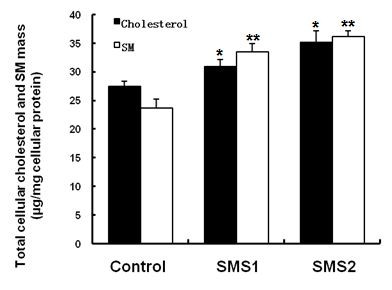
**Determination of the cellular SM and cholesterol content**. Values shown are means ± SD (*n *= 3), *P < 0.05; **P < 0.001 compared to control (Huh7-tTA).

**Figure 4:**
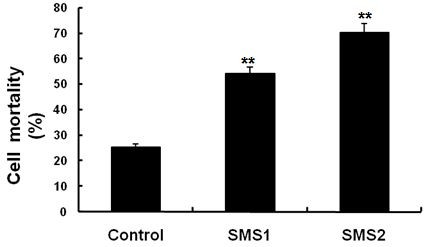
**Lysenin-mediated cytotoxicity**. Values shown are means ± SD (*n *= 4), *P < 0.001 compared to control (Huh7-tTA).

As shown in Figure 3, SMS overexpression significantly increased intracellular levels of SM (*P *< 0.001). Because cellular SM levels represent SM levels in all cellular membranes, including the plasma membrane, endoplasmic reticulum, and Golgi complex, we still do not know whether SMS1 or SMS2 overexpression has an effect on SM levels specifically in the plasma membrane. Lysenin is a recently discovered SM-specific cytotoxin that recognizes SM only when it forms aggregates or microdomains [24]. To investigate the effect of SMS1 or SMS2 overexpression on the formation of these microdomains, we tested both SMS overexpression cell lines for their sensitivity to lysenin-mediated cytolysis. As indicated in Figure 4, both cells showed significantly more sensitive to lysenin-mediated cytolysis than control cells (*P *< 0.001).

There is evidence to suggest that sphingomyelin and cholesterol are linked inside the cells [25]. We therefore reasoned that the accumulation of SM in the cells might increase cellular cholesterol levels. To test this hypothesis, we measured cellular cholesterol levels and found that they were significantly increased in both SMS1 and SMS2 cells compared to control cells (Figure 3; *P *< 0.05).

**Figure 5:**
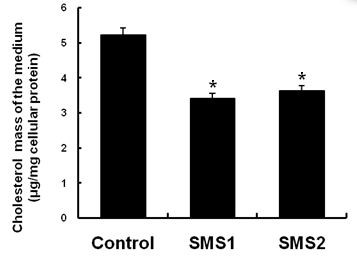
**Determination of the cholesterol concentrations in the medium**. Values shown are means ± SD (*n *= 3), *P < 0.05 compared to control (Huh7-tTA).

**Figure 6:**
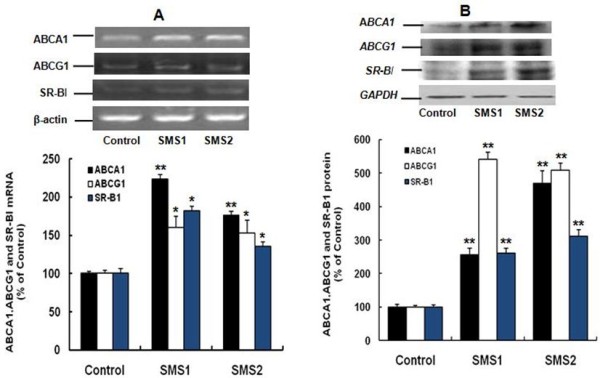
**The expression of ABCA1, ABCG1 and SR-BI**. **A**. RT-PCR analysis; **B**. Western blot analysis. Values shown are means ± SD (*n *= 3), *P < 0.05; **P < 0.001 compared to control (Huh7-tTA).

**Figure 7:**
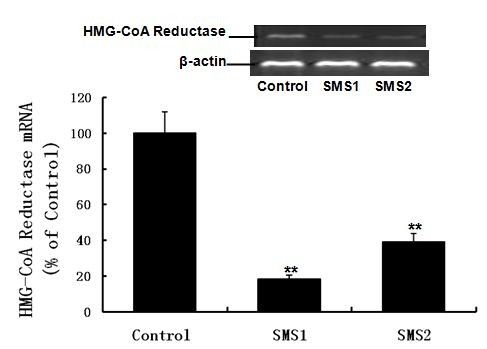
**mRNA analysis of HMG-CoA reductase by RT-PCR**. Values shown are means ± SD (*n *= 3), **P < 0.001 compared to control (Huh7-tTA).

We next sought to determine whether overexpression of SMS would decrease cholesterol secretion. The results showed that the contents of cholesterol in the cell culture medium were significantly decreased in both SMS1 and SMS2 overexpressing cells (Figure 5; *P *< 0.05). We then measured ABCA1, ABCG1, SR-BI mRNA and protein levels, as well as HMG-CoA reductase mRNA levels. As shown in Figure 6, SMS1 and SMS2 overexpression can upregulate ABCA1, ABCG1, and SR-BI mRNA and protein levels and downregulate HMG-CoA reductase mRNA levels, as compared with controls (Figure 6, 7; *P *< 0.05 and 0.001, respectively).

**Figure 8:**
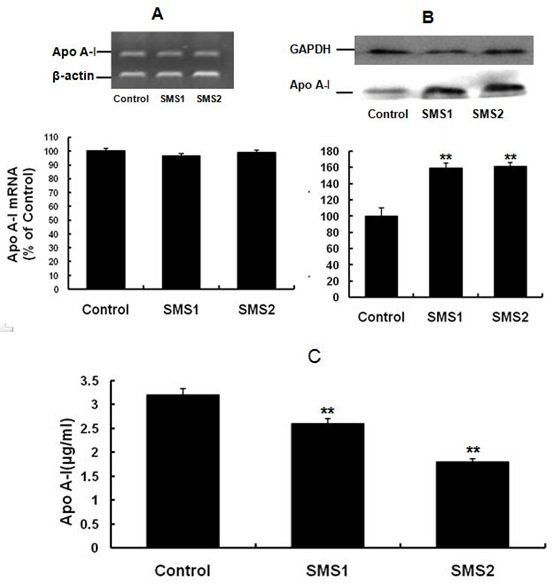
**Analysis of Apo A-I in the cells and medium**. **A**. Apo A-I analysis in the cells by RT-PCR; **B**. Apo A-I analysis in the cell by Western blot; **C**. Apo A-I analysis in the medium by ELISA. Values shown are means ± SD (*n *= 3). **P < 0.001 compared to control (Huh7-tTA).

To investigate the relationship between SMS overexpression and lipoprotein metabolism, we measured Apo A-I levels inside the cells and in the cell culture medium. As shown in Figure 8, SMS overexpression significantly increased cellular Apo A-I content (Figure 8B*; P *< 0.001), which might be due to decreasing Apo A-I secretion (Figure 8C; *P *< 0.001). Apo A-I mRNA expression between SMS cells and control cells were not significantly different (Figure 8A; *P *> 0.05).

## Discussion

Previously, we had confirmed that overexression SMS1 or SMS2 can accelerate development of atherosclerosis in mice. But the studies were in mice[20], to further investigate the effect of SM on the atherosclerosis in human cells, in the present study, we demonstrated that Huh7 cells with higher SMS activity 1) had increased cellular SM and cholesterol content and decreased cholesterol secretion; 2) had increased cellular Apo A-I content and decreased Apo A-I secretion, but normal Apo A-I mRNA expression; and 3) had increased expression of ABCA1, ABCG1 and SR-BI mRNA and protein levels as well as decreased expression of HMG-CoA reductase mRNA. Thus, SMS activity is closely related to cholesterol metabolism.

In this study, we demonstrated an interesting phenomenon: SMS overexpression-mediated SM content changes paralleled the cholesterol content changes in the cells. The interaction of SM, cholesterol, and glycosphingolipid drives the formation of plasma membrane rafts [8]. Rafts are formed in the Golgi apparatus and are targeted to plasma membranes, where they are thought to exist as floating islands within the sea of bulk membrane [26]. Up to 70% of membrane SM is found in rafts [27]. The relative proportions of both SM and cholesterol appear critical for raft stability because depletion of cholesterol or supplementation of the SM pool abolishes or (re)establishes, respectively, the detergent insolubility of the domains and their sorting capacity [28-30]. As demonstrated in artificial membrane systems, cholesterol facilitates the formation of sphingolipid-containing microdomains [31]. Ito et al. [25] reported that the digestion of membrane by extracellular sphingomyelinase (SMase, a SM hydrolase) increased the incorporation of cellular cholesterol into generated HDL and that this increase was diminished by supplement of endogenous or exogenous SM to the cells. Patients with Niemann-Pick disease (NPD-B) can not hydrolyze SM because of defective SMase, which results in the deposition of a large number of SM and cholesterol in the liver and nervous system [32]. Our results imply that SM and cholesterol in the rafts might closely interact with or anchor one other.

Our results also showed that SMS overexpression-mediated cholesterol accumulation (Figure 3) was not related to increased production, because HMG-CoA reductase mRNA expression was downregulated (Figure 7). A reasonable explanation for this result follows: 1) elevated SM can increase cholesterol sequestration; 2) Apo A-I is the acceptor of cellular cholesterol efflux, so decreasing the secretion of Apo A-I might result in reduction of cholesterol efflux; and 3) as an HDL receptor, SR-BI in hepatic cells involves selective HDL cholesterol ester uptake and cholesterol efflux[3,7] and increasing SR-BI expression (Figure 6) might promote cholesterol uptake and cholesterol efflux, but the mass of upake were more than the efflux, Probably, cholesterol in Golgi complex or endoplasmic reticulum (ER) was increased along with SM. Our previous study also showed that SMS overexpression led to upregulation of SR-BI in vivo [20]. Apo A-I plays an important role in anti-atherosclerosis. There is a negative relationship between plasma HDL-C levels and atherosclerosis, and Apo A-I is a major protein component of HDL [2,4]. Because Apo A-I mRNA expression had no change, increased cellular Apo A-I content in SMS-overexpressing Huh7 cells might be due to decreased Apo A-I secretion (Figure 8).

Downregulation of HMG-CoA reductase expression in SMS overexpressed cells reflects a feedback inhibition mechanism by elevating cellular cholesterol content (Figures 3 and 7). Upregulation of ABCA1 and ABCG1 expression in SMS overexpressing cells might be a self-protective positive feedback mechanism (Figure 6), consistent with a reported study that showed that in epoxycholesterol treated macrophages, where an increase in cholesterol synthesis led to the upregulation of ABCA1 and ABCG1 [33].

The deposition of cholesterol in plaques is one of the characteristics of atherosclerosis. Our previous studies showed that SMS overexpression resulted in changes in plasma lipid and lipoprotein metabolism and increased the atherogenic potential in mice [20]. All evidence indicates that the inhibition of SM biosynthesis might be a new pathway for the treatment of AS and that SMS might become a potential target for AS treatment.

## Conclusions

Cellular SM levels are positively related to cellular cholesterol levels and SMS overexpression-mediated cellular SM content changes are related to cellular Apo A-I content and secretion. SMS might be a potential target for the treatment of AS.

## Competing interests

The authors declare that they have no competing interests.

## Authors' contributions

YNL screened the monocloned cell lines and carried out RT-PCR, Western blot, ELISA analysis and Sphingomyelin synthase activity assay. TBD measured the cell mortality of lysenin to huh7 cells. JBD and YL participated in measuring the content of SM and cholesterol. MPW conceived the study, participated in experimental design, and drafted and revised the manuscript. All authors have read and approved the final manuscript.
